# “They created a team of almost entirely the people who work and are like them”: A qualitative study of organisational culture and racialised inequalities among healthcare staff

**DOI:** 10.1111/1467-9566.13414

**Published:** 2021-12-06

**Authors:** Charlotte Woodhead, Nkasi Stoll, Hannah Harwood, Team TIDES Study, Obrey Alexis, Stephani L. Hatch

**Affiliations:** 1 Department of Psychological Medicine, Institute of Psychiatry, Psychology & Neuroscience, King's College London, London, UK; 2 ESRC Centre for Society and Mental Health, King’s College London, London, UK; 3 Department of Nursing, Faculty of Health and Life Sciences, Oxford Brookes University, Oxford, UK

**Keywords:** bullying, discrimination, ethnicity, Healthcare staff, qualitative, race

## Abstract

Racially and ethnically minoritised healthcare staff groups disproportionately experience and witness workplace discrimination from patients, colleagues and managers. This is visible in their under-representation at senior levels and over-representation in disciplinary proceedings and is associated with adversities such as greater depression, anxiety, somatic symptoms, low job satisfaction and sickness absence. In the UK, little progress has been made despite the implementation of measures to tackle racialised inequities in the health services. So, what is it about the health service organisational context which shapes and maintains such inequities, and what role does discrimination, bullying and harassment play? Drawing on qualitative interviews with 48 healthcare staff in London (UK), we identify how micro-level bullying, prejudice, discrimination and harassment behaviours, independently and in combination, exploit and maintain meso-level racialised hierarchies. Within teams, the high diversity–low inclusion dynamic shaped and was perpetuated by in- and outgroup inclusion and exclusion processes (including “insidious dismissal”) often employing bullying or microaggressions. These were linked to intersecting factors, such as race, ethnicity, migration, language and religion, and could increase segregation. For racially and ethnically minoritised groups, ingroup maintenance, moving teams or leaving were also ways of coping with organisational inequities. We discuss implications for tackling racialised workplace inequities.

## Introduction

International evidence indicates that employees from marginalised social groups experience discriminatory workplace exclusion, impacting job access, career progression, disciplinary actions and support networks (Mor Barak, 2015; Offermann et al., 2014). Racially and ethnically minoritised people report exposure to “microaggressions” (Sue et al., 2007), racist stereotyping, prejudice and overt unfair treatment (Mor Barak, 2015; Offermann et al., 2014). These experiences are associated with low employee satisfaction, engagement and greater staff turnover (Deitch et al., 2003; Nunez-Smith et al., 2009). Similarly, workplace bullying and harassment adversely affect employee morale, productivity, mental health and wellbeing (Kline, 2019; Rhead et al., 2021).

Racial and ethnic minority groups are over-represented in the NHS workforce compared with the working age general population, particularly Asian (10.7% versus 7.2%) and Black staff groups (6.5% versus 3.4%) (NHS Digital, 2021). Approximately 14% of NHS staff are from over-seas (26% in London), with Indian, Filipino and Irish migrant groups commonly represented and particularly among nurses (Baker, 2020). Yet, compared with their colleagues from White majority groups, migrant and racially minoritised nursing groups disproportionately occupy lower paid and junior roles (NHS Digital, 2021). Moreover, they report higher levels of racial discrimination and harassment (Likupe, 2015; Likupe & Archibong, 2013), exposure to negative assumptions about their clinical abilities (Batnitzky & McDowell, 2011), are subject to greater workplace disciplinary proceedings (Likupe & Archibong, 2013), and greater formal questioning of their fitness to practice (West et al., 2017). These experiences are associated with emotional problems, somatic symptoms, low job satisfaction and long periods of sickness absence among this workforce (Rhead et al., 2021).

In response, the Workforce Race Equality Standard (WRES, 2021) was mandated in England in 2015. This requires NHS organisations to demonstrate improvement against nine indicators of racialised inequities, though little progress has been reported since 2016 (WRES, 2021). As with discrimination, bullying and harassment are systemic and multi-level and social phenomena (Came & Griffith, 2018; Kline, 2019), it is necessary to examine the processes through which the institutional and workplace context shapes these phenomena to inform approaches to interventions. This study addresses the following questions: (1) How do healthcare staff describe their workplace context? (2) How, and through which social processes does this context influence experiences of discrimination, bullying and harassment? (3) How might this account for the adverse experiences of racially minoritised healthcare staff? (4) How are these aspects of workplace context established, maintained, perpetuated and resisted?

## Theoretical Framework

We draw on relevant concepts from critical race theory (CRT) and from workplace diversity and inclusion literatures. CRT draws on theory, experiential knowledge and critical consciousness to argue that racism is systemic and embedded into institutional structures (e.g. health, housing, work) and that interrelations between race, racism and power embed racialised inequalities (Delgado & Stefancic, 2001). Intersectionality theory, another key relevant aspect of CRT, examines discrimination, privilege and power at the intersections of multiple individual, social and political identities (Crenshaw, 1991). This allows for a more nuanced understanding of workplace racialised inequities (e.g. capturing experiences at the intersections of migration status and race/ethnicity).

Workplace diversity perspectives emphasise how intergroup biases may affect the experience of minoritised staff groups (Turner et al., 1987). Informational/decision-making perspectives emphasise differences in attitudes, opinions, work styles and knowledge (Van Knippenberg & Schippers, 2007). These perspectives underpin positive effects of workgroup diversity on performance through elaboration of task-relevant information and constructive reflection (van Knippenberg et al., 2004).

Finally, the concept of workplace inclusion refers to whether individuals feel that they belong and are valued for their unique attributes and contributions (Shore et al., 2011). Inclusion involves feeling respected and valued, psychologically and physically safe to be authentically themselves at work and share divergent views and opinions, even if those differ from dominant cultures. It also means being involved as insiders with access to key resources, having decision-making influence and perspectives that are listened to (Shore et al., 2018).

## Methods

### Design and setting

This qualitative interview study uses data from the mixed methods Tackling Inequalities and Discrimination Experiences in health Services (TIDES) study. TIDES aims to identify what is generating, perpetuating and possibly mitigating inequalities in health services (www.tidesstudy.com). Phase one was London-based, involving healthcare staff (student and qualified nurses/midwives, healthcare assistants (HCAs)) recruited from 33 of the 34 London trusts (geographically based organisations providing patient care including hospital and community services). Forty-five per cent of the NHS workforce in London identifies as a racial or ethnic minority group, over a quarter (26%) are migrant staff (non-UK born), and London consistently ranks as the worst performing region in England for many WRES indicators (WRES, 2021).

### Participants and sampling

Qualitative data were collected from TIDES phase one survey participants (*n* = 931, detailed in Rhead et al. (2021). We purposefully sampled those who had agreed to be re-contacted, including racial and ethnic minority and White British staff at different levels of seniority (organised in the NHS by salary levels, “pay bands”).

### Data collection

Data collection occurred between January 2019 and February 2020, just prior to the UK COVID-19 pandemic. Potential participants received an e-mail invitation and up to three further contacts. Those expressing interest were sent information including details on confidentiality and withdrawal. Participants were assigned a unique ID number, gave informed consent prior to interview and were offered £15 shopping vouchers for their time.

An experienced qualitative researcher conducted semi-structured interviews lasting 45–60 min in person or by telephone dependent on participant preference, which were audio-recorded with consent. Topic guides were developed through examination of literature and engagement with healthcare professionals at participating trusts. Topics covered experiences of and witnessing workplace discrimination, bullying, and harassment, reporting processes, and training and support. Data were transcribed verbatim, removing identifying information.

### Data analysis

Data were inductively thematically analysed (Braun & Clarke, 2012) by a racially and ethnically diverse research team. Following data familiarisation, transcripts were descriptively coded by four researchers to develop an initial coding framework, refined through iterative discussion and coding rounds. Data were imported into NVivo (QSR International Pty Ltd, 2018) to support data management and the coding framework applied to all transcripts. Supported by research memos, discussion and visual mapping, the team actively sought themes reflecting relevant key patterns within and across interviews. These were continually checked and refined against transcripts, looking for patterns by participant characteristics and for similarities and differences within and across datasets. Themes were defined, described and labelled, and patterns were discussed.

Quotes are labelled with participant race/ethnicity, migration status and seniority level (ascertained from TIDES survey responses). To maintain anonymity, race and ethnicity are presented as Asian, Black (including Black African, Black Caribbean and other Black groups), White British, White other or any other ethnic group, based on self-reported Office for National Statistics classifications (Office For National Statistics, 2021). We grouped seniority levels into student nurse, HCAs (pay Bands 2–3) / nursing associate (Band 4), entry-level qualified nurse/midwife (Band 5) and mid- or senior-level qualified nurse/midwife (Bands 6–8). This serves to maintain anonymity and reflects the over-representation of racial and ethnic minority staff groups at Bands 1–5, with considerable racial disparities in progression beyond entry-level (Band 5) pay grades.

### Ethical approval

Ethical approval was granted by the King’s College London Research Ethics Committee for Psychiatry, Nursing and Midwifery (HR-17/18-4629; RESCM-19/20-4629; RESCM-20/21-4629) and NHS Health Research Authority (18/HRA/0368).

## Results

### Sample

Forty-eight healthcare professionals representing 17 of the 33 participating trusts were recruited. Participants worked across various specialties, roles and pay bands. The majority of the sample identified as female (85%), two fifths were White British, over a quarter were from Black groups, and over a third were migrant staff. Over two fifths were student nurses/midwives, a fifth at entry-level (Band 5) posts, and just under a quarter were mid or senior nursing/midwifery staff (eight band 6 and 7 nurses, and three band 8 nurses). Of note, several student nurses also had HCA experience (Table 1).

**Figure 1:**
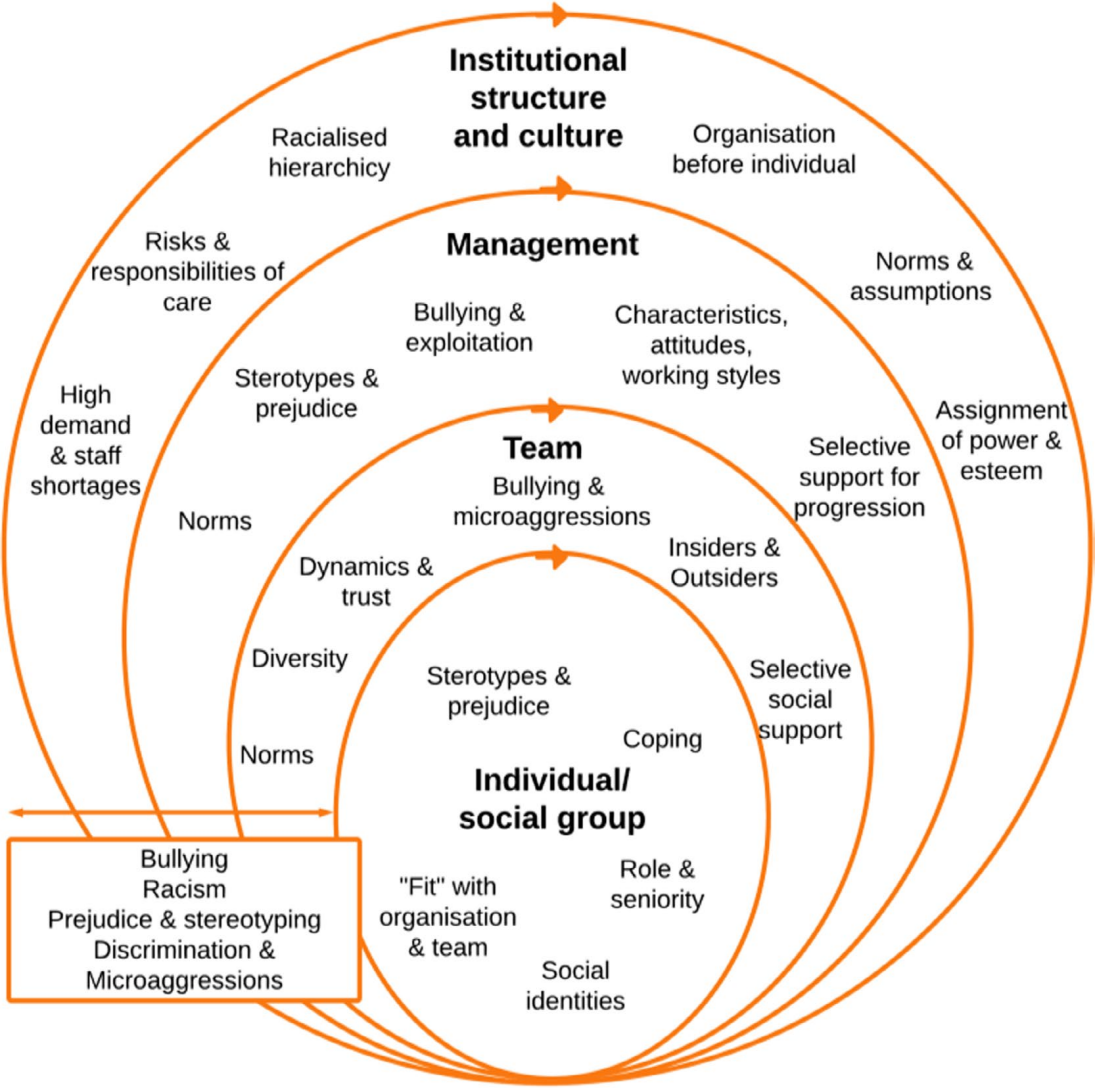
Factors shaping and maintaining racialised inequalities in healthcare organisations

Three key interacting contextual features shaped the social processes influencing the experience and perpetration of discrimination, bullying and harassment among racial and ethnic minority staff: hierarchical organisation, pressurised work environment and high diversity–low inclusion. We briefly describe how the pressurised environment and hierarchical organisation shaped the conditions for discrimination, bullying and harassment, and their implications for staff experience. We then identify how the disproportionate representation of racial and ethnic minority staff groups at lower-banded roles, alongside prejudice and stereotyping, was shaped by, maintained and perpetuated workplace inequities. Last, we examine how the workplace environment contextualised and shaped a culture of high diversity but low inclusion operating within and between hierarchical levels, increasing segregation through in- and outgroup processes (see Figure 1).

### Hierarchical organisation and workplace pressures

Participants unanimously described the health service as hierarchical, with responsibility and risks associated with patient care and decision-making distributed by “rank.” This was primarily seniority-based, with sub-divisions within and between levels associated with role, perceived experience and expertise, duration in the team, and permanent or temporary status (e.g. agency or bank staff). At the peaks of the clinical hierarchy were senior doctors and consultants while the lowest levels were occupied by students, staff without patient-facing roles or hired by an external agency.

We all know that the doctors are gods, we perceive. Then you’ve got the matron, and then you’ve got the ... and it comes down, and being an HCA [Health Care Assistant], you are the end of that.

*Black, non-migrant, student nurse/midwife*

Lower-banded positions were described as involving roles that are vital, yet also perceived as more menial, or requiring greater direct patient and family contact. Further up, qualified nurses described greater line management roles and responsibilities for clinical decision-making.

I think the nurses get the brunt of it yeah, um. But then that has a knock-on effect on the patients because they’re so stressed and they’re like, “I haven’t got time to um deal with this situation, and then maybe the healthcare assistants will deal with it because then they’ll be like “healthcare assistants, de-escalate this situation or deal with this situation with the patient, I haven’t got time I need to be in the office, I need to do this I need to do that. Because the manager’s on my case.”

*Asian, non-migrant, student nurse/midwife*

The social currency of this hierarchy was power, decision-making latitude, autonomy and respect. This currency afforded “value”—assigned or perceived—in relation to their status-based role, clinical opinion, contribution to the team and respect. Lack of value was particularly reported by or on behalf of the lower-banded staff but also by qualified nurses.

Wherever you are within the hierarchy there’s always someone above you, there’s always this feeling of, erm, not being valued. Err, so for example, err, I don’t feel like nurses feel valued by the medical teams. Erm, and I think that Healthcare Support Workers don’t feel valued by nurses.

*White British, non-migrant, student nurse/midwife*

Perceiving lack of recognition, reward, being “one of a number” or replaceable, as well as unsupported in personal career development, exacerbated negative feelings about value, esteem and job satisfaction.

I think because of the hierarchy culture, erm, I think it makes it quite easy for some people to, erm, that – there’s almost a lack of appreciation for what other roles do within the organisation. Erm, and I think when there is that, people tend to forget how hard others are working, or their, you know, their contribution they can make to a patient experience.

*White British, non-migrant, student nurse/midwife*

I think because you feel one of a number, you don’t feel literally specialised in your role, you’re just doing a generic job in a way. And I think that’s probably the key point that you are, you do just feel one of many and you could be easily replaced.

*Other ethnic group, non-migrant, qualified nurse/midwife*

Workplace pressures were experienced differently across hierarchical levels. Pressure was described in terms of heavy workloads and staff shortages as well as the demands and expectations of patients, families and management. This was intensified by the risks, responsibilities and possible repercussions of caring for vulnerable patients.

A lot of our time is spent on documentation and the number of patients, that’s too many for you to give enough for every patient, like seven patients, it’s too much. […] And sometimes there will be shortage of staff or sometimes you will not actually be understaffed but we have agency staff who are working for the first time, so they need a lot of support.

*Black, migrant, qualified nurse/midwife*

Several HCAs, and some qualified nurses/midwives, described the physical toll of such pressures alongside a culture of expectation to put workplace demands before their own health and wellbeing.

You would not have time to go to the toilet to pass urine or you wouldn’t have time to drink. So, you would treat the, that’s what happens actually, you would treat the dehydration for the patient but as a nurse you would be dehydrated yourself.

*White other, migrant, qualified nurse/midwife*

I think in the NHS especially, everyone sometimes expects you to work above and beyond really without having a support. So, sometimes there is an assumed perspective of, you are going to stay late and that’s just the way it is.

*Black, non-migrant, student nurse/midwife*

Cooperation and collaboration were key to performing under workplace strains but could also be compromised in stressful working environments. Participants placed great importance on being able to rely on and trust colleagues. Positive team dynamics strongly influenced staff wellbeing but was described as varying between workplaces.

If you think about the environment, you know, you’re looking after sick patients… You’ve got… you’ve got anxious, angry parents; you’ve got, you know, all these pressures, not enough staff. There’s, you know, so much going on and you don’t get a break, etc., and I think all that adds to the heightened like environment…it can cause people, on stressful days, to really come… you know, get a bit short-tempered and stuff… Unfortunately, I think it happens more in a dysfunctional team than it does in a functional team.

*Asian, non-migrant, qualified nurse/midwife*

Such experiences were contextually variable and several participants highlighted examples of working with a “good team.” This not only helped them cope with workplace pressures, but also protected against hierarchical exploitations of power.

A good ward is one that does treat every person as equally as a person and as a valued member of the team[…]on my ward it is quite good that they’re even - you know, like, cleaning staff and kitchen staff and people who are assigned to work on the ward, who aren’t even necessarily hired by the ward, but will, kind of, count them as part of the team and invite them to ward events and stuff and talk to them and treat them like a part of the team. But there are places where, I think, those, kind of, surrounding staff are somewhere, like, ignored.

*White British, non-migrant, qualified nurse/midwife*

### Exploiting the vertical hierarchy

The workforce hierarchy and associated devaluation of lower-banded roles could extend into perceived or actual abuses of power, with adverse effects on job satisfaction, engagement and intentions to leave.

I’ve seen people, like, managers calling people out publicly in front of a ward, seeming to almost embarrass them on purpose when what they did wasn’t really that bad and you think it’s just because they don’t really like that member of staff, and almost wanted to embarrass them.

*White British, non-migrant, qualified nurse/midwife*

I had a terrible experience with someone senior in the organisation who made my life hell basically, and it didn’t end well, and I agreed to leave the organisation at the end of the year.

*Black, migrant, senior qualified nurse/midwife*

As with workplace pressures, bullying and harassment could also affect patient care and experience, directly (affecting capacity to provide optimal patient care or clinical decisions) and indirectly (by creating a negative working environment).

If the team morale is down, people are going to be ill more, and people are going to be dreading coming into work and then actually, that probably will lead to mistakes because if you’re going to be anxious, stressed, it’s just a cycle.

*Other ethnic group, non-migrant, qualified nurse/midwife*

While division of labour may be inherent to the hierarchy and necessary to organisational function, it could also be experienced as exploitative when requests to carry out tasks were made in ways perceived as wielding power and when staff felt unreasonable pressure was placed on them.

In my actual workplace you can tell that if somebody doesn’t like you by their difficulty or how complex your patients are on the day. It seems like, urm, if somebody doesn’t like you then whoever is allocating work, if they don’t like you, it will reflect in your workload on the day.

*White other, migrant, HCA/nursing associate*

### Maintaining a racialised vertical hierarchy: discrimination, prejudice and harassment

While workplace strains and exposure to abuses of power were described by staff across different racial or ethnic groups, the hierarchy itself was inherently racialised with lower levels disproportionately occupied by racially and ethnically minoritised groups. Participant accounts indicated that they were unduly exposed to adversities related to workplace pressures and hierarchy-based bullying and exploitation.

When you went into the staff room, it was really tiny anyway but it’s only… and our nurses were all White nurses. And all the White nurses and the HCA was fine. There was another two admin staff that were, um, of White English background and they were in there. And everything was fine. But all the cleaners, any admin, like, other HCAs, the housekeeper, it felt like we weren’t allowed in there. Even when I went in to sit down, I felt like I wasn’t… Like, what am I doing here, sort of thing. Like, you guys don’t belong here in the staff room.

*Asian, non-migrant, student nurse/midwife*

When nurses return back to work, like, after sickness, usually they’re given, um, a more lenient workload. Like, they, they’ll have, like, patients that have, like, less interventions or, like, less heavy you just call it. Um, and then, um, then if it’s a BME [Black, Asian and Minority Ethnic group], sorry, nurse, it’s not… like, they’ll still get the same workload. Like, they wouldn’t be as sympathetic.

*Asian, non-migrant, student nurse/midwife*

Importantly, participants highlighted ways in which negative racial prejudices and stereotypes amplified such hierarchical inequities. At individual and team levels, such appraisals were systematically detrimental to the treatment, appraisal and support for racially and ethnically minoritised staff.

Sometimes, like, based on, based on stereotypes, some of my colleagues, when we have healthcare assistants, they have, like, there’s a stereotype that the healthcare assistant, the Black healthcare assistant are lazy. So, there are like… I don’t want to say bully them. Because it’s a bit, like, hard. But they will be more like, can you do that, and can you do that and can you do that? So that they will not sit down that much.

*White other, non-migrant, qualified nurse/midwife*

We have more, I would say, Black Afro-Caribbean members in our team, actually […] we are an overstretched team, and my colleagues have made comments of, we’re not given the same … I don’t know the truth to this, but I just know we are an over-stretched team and other wards do have … probably may have better support than we do.

*Black, non-migrant, student nurse/midwife*

These phenomena reinforced the division of power, “reminding” people of their position. They could also act more explicitly to maintain the racially and ethnically inequitable distribution of staff by influencing progression. Vertical progression was described as dependent on being permitted or supported to access career-enhancing training by those with greater power and therefore vulnerable to exploitation.

On the one hand, selective exclusion and ingroup favouritism seemingly unrelated to race or ethnicity were described across participant groups; for example, linked to perceived competitive threat or nepotism.

When I think of the people who were belittling or undermining or I would say bullying as well in a way, in hindsight, I think they were the ones that felt threatened by the more, um, junior members of the team who had a little bit of spark and interest and, you know, drive to move forward and maybe they felt their positions were threatened or they were trying to put people back in their place.

*White other, migrant, qualified nurse/midwife*

You tend to get groups that are very close to the mangers and when they want to go and do something or do a course, they get booked on it really quickly. Or they want to get annual leave at a certain time and get first priority.

*Other ethnic group, non-migrant, student nurse/midwife*

However, participants’ accounts also illustrated ways in which discrimination, prejudice, bullying and harassment served to maintain the hierarchy and its racialised disparities. Staff from racially and ethnically minoritised groups reported witnessing, experiencing and anticipating unfair treatment. This impacted progression directly but also indirectly, by fostering an accumulation of fatigue and resignation, deterring attempts to progress and further perpetuating the status quo.

I know some people have the view of, and it is seen more when you get to higher levels of practice that BAME [Black, Asian and Minority Ethnic] members of the team don’t feel as valued, they don’t feel like they’re given promotional opportunities as much as their White or Caucasian counterparts.

*Black, non-migrant, student nurse/midwife*

Some just anticipate because obviously they’ve heard their friend went and didn’t manage and I think we do suffer from that a lot, where we just like, it’s just not worth the hassle. We’ve seen too many people go through it and not get there, so we can’t be bothered […] if you and speak to a lot of staff now, that is the morale that a lot of them have, they’re just, ‘we know we’re never going to get this, we know what’s going to happen, so what’s the point?

*Black, non-migrant, senior qualified nurse/midwife*

Prejudicial views and discriminatory language also underpinned opinions expressed by some (White British) participants that unequal progression was either the fault of underrepresented groups themselves or down to personality clashes rather than attributable to prejudice.

I think one of the reasons is there’s an element of self-choice. I think also they don’t believe they can do it. That’s either cultural, either their schooling, either their country of origin - we have a real problem with Filipinos [...]ín the Philippines it’s much wider apart, so women already are feeling less valued. And also, they, they are much more, um, subservient. And especially in, they have a pecking order in things. they’ll always back down if someone is more senior.

*White British, non-migrant, senior qualified nurse/midwife*

With student nurses I worked with one nurse and I think they saw her as a more argumentative kind of bolshie person. She was a, ugh, her accent was quite London street. So I don’t know whether they honed in on that, but they treated her very differently to the other student nurses. I don’t think it was race because everybody on that ward was a different race. So I can’t think it was because of that, um. Maybe it was just a personality clash that one. I’m not sure.

*White British, non-migrant, student nurse/midwife*

These stereotypes also led to unfair professional judgements and misinterpretations about racial and ethnic staff members. This could affect progression directly by constraining support and positive appraisal.

Most of the people in senior posts, they are not culturally competent. They are good at their job but when you do not understand certain things, for example, observe an Afro-Caribbean person. Even when they’re having a chat. The voice is so strong. Comes across like it’s a fight. It sounds like it’s aggression even when they are having a laugh. So, that sunshine which is common in the European voices, is not usually in the African culture or the Caribbean culture except for those people that are probably born here, who’ve picked up those things. So, you know, most people, when you’re talking to them, they can’t look in your eyes because it’s a sign of respect, especially where you are their boss at work. No matter what you do, they can’t look in your eyes. But in this culture, if you can’t look into people’s eyes it looks like you’re lying. So, it takes somebody that knows much more than mashed potatoes and gravy to actually decipher this thing and be able to judge right.

*Black, migrant, student nurse/midwife*

Finally, the racialised hierarchy was also more insidiously maintained by enactments of resistance to racially minoritised staff occupying more senior positions (thus “contradicting” the racialised hierarchy norm) through microaggressions and more overt discrimination.

We normally split our team and you will have a Band 6 leading them and then, you will have like, four nurses, four healthcare assistants and there was one particular one that said, “I’m not going to work with her”, “Why?”, “ I don’t understand her English” […] he refused, and he was telling her in her face, “your English is ridiculous. I don’t want you to be my team leader.”

*White other, migrant, qualified nurse/midwife*

Similar to the impact of sustained disadvantages in career progression described above, participants portrayed how chronic exposure to and battles against these everyday undermining experiences had an accumulative wearing effect on those reaching more senior levels.

Nothing I did was right. It was tough. I will have two or three invites in my inbox for meetings at different parts of the hospital. I will be humiliated in meetings. I will be undermined if I was trying to do some work, for example, with my team, I will be undermined, and it was just difficult. In hindsight I should have left, but I found in the statistics of BME staff that we put up with a lot more. So, I worked and worked and worked my bones off, and then I was accused of random stuff which was not true, and I which I knew wasn’t true and I should have fought a bit more but at the time I was broken and I just didn’t want to work there anymore. Black, migrant, senior qualified nurse/midwife. We next examine how dissimilarity on racialised dimensions (e.g. language, accent or visible markers of religion) amplified in and outgroup inclusion and exclusion processes horizontally *within* hierarchical levels.

### Horizontal hierarchies: insiders and outsiders

Participants’ accounts indicated that being the “right fit” to the prevailing workplace culture depended on the nature and composition of the workforce. For example, determined by intersecting dimensions of demographic diversity (race, ethnicity, religion, migration status), perceived functional attributes (e.g. duration in the team, expectations around working styles, competence), as well as attitudes, personality, and ways of socialising.

If it’s a well-known team and they’ve been working together for years, a new person coming in, they’re not … I want to say, they give you a hard time. They’re not warming. You really have to make your presence known basically […] I think they close ranks. I don’t think it’s about earning respect. I think it’s like when you’ve got a foreign object in your body and your body is fighting it until it gets used to it.

*Black, non-migrant, student nurse/midwife*

However, these varying criteria of “fit” were all vulnerable to prejudice and stereotypes and demonstrated lack of inclusion, such as intolerance of cultural differences in working styles.

I’m White British…and so from where I was standing it seemed like the African staff were more, like, disciplinarian and, kind of, conservative, and I felt uncomfortable with that. But I’m guessing there’s, like, cultural differences and beliefs that I don’t fully understand, that meant that that made sense for them. But then it’s, like…like, so to me having rules, like, that the patients have to stand in a line and not speak before they can get their medication seems, like, pointless and it’s, like, well this isn’t a…this isn’t the Army.

*White British, non-migrant, student nurse/midwife*

She [a colleague] was working on a ward with a lot of, um Black African nurses and she said um, she said that she didn’t like working with them and I asked why, and she said that they had a lot of attitude and then she used the phrase “Black attitudes” and she said similar sort of things to what the patients would say. She said they were bolshie. That they’re superior, um. They were non-communicative and I think she even said something like, that they were ignorant […] she was one person and it’s not a view that I’ve noticed in a lot of other people, especially not that strongly, but I would say there was this attitude of, um, yeah towards, towards West Indian or African nurses. *White British, non-migrant, student nurse/midwife*

After working for a day or two, my manager came in and said that he doesn’t want Blacks there.

*Black, migrant, qualified nurse/midwife*

Depictions of diversity in relation to race, ethnicity and migration status varied according to the team composition and the attributes of the person perceiving it. For instance, in line with information and decision-making perspectives, workplaces comprising staff from multiple different racial or ethnic groups were perceived by many as positive working environments beneficial to patient care.

I had one placement where it had a really positive approach with diversity ‘cos like, there was just, like, within that team you had…there was, like, people from the Caribbean, from the Middle East, like, White British people, people from Mauritius and, like, it would be…people were just, kind of, like, share what they…their, like, kind of, what they knew and their cultural backgrounds they, kind of, would use that in their work, like, if someone had a patient who was, like, Nigerian or something there would always be someone on the team who would be, like, “Oh, the reason he’s behaving that way, that’s not a mental health issue that’s, like, a cultural thing cause in our culture we do this and this.” So that team it, kind of, having a mixture worked really well.

*White British, non-migrant, student nurse/midwife*

In contrast, diversity was portrayed more negatively in relation to inter-ethnic group tensions or hierarchies and where there was a clear divide between racial and ethnic groups, particularly if this involved an uneven split or an inversion of “traditionally” minority and majority groups.

I think it’s more, if you’ve only got one or two different groups then sometimes you end up with, like, everyone, kind of, joins sides with one group or the other and you, kind of, end up with, like, you know, like, there’s, sort of, one social group that’s, like, all Black and one that’s all White and it’s, like, kind of, a bit odd.

*White British, non-migrant, student nurse/midwife*

This type of segregation maintained horizontal hierarchies defined by in- and outgroups by two distinct processes, “insidious dismissal of outsiders” and “resistance and coping.”

### Maintaining horizontal hierarchies

#### Insidious dismissal of outsiders

Insidious racially motivated inclusion and exclusion processes were described in which people were influenced to leave (“insidious dismissal”) or supported to stay within a particular workplace. This contrasted with the more explicit bullying and discriminatory behaviours which maintained vertical hierarchy.

There are wards that, you know, places can get quite, kind of, clicky and get quite a specific culture and, you know, people get their friends on and, you know, bring people in and then if someone doesn’t like a member of the team, they’ll just treat them badly and then they will eventually leave, so that means you’re just creating this kind of, you know, survival of, like, them people where, you know, everyone else will leave if they’re not the, kind of, people that they want until they, you know, have created a team of almost entirely the people who work and are like them and then it’s horrible for anyone who goes and isn’t exactly like that.

*White British, non-migrant, qualified nurse/midwife*

Some certain kind of people, they will support more their people. Like, one place I used to work had a lot of Filipinos. So, some of the band sixes were Filipinos. And then when in the shift there were more Filipinos, they will support them more compared to how much they will support us.

*White other, non-migrant, qualified nurse/midwife*

They’d go out after work with the White English, like, they’d include her in as well. Like, they’d go out for drinks whatever. But the Black student nurse was never… wouldn’t be invited.

*Asian, non-migrant, student nurse/midwife*

#### Coping

Participants expressed how racial and ethnic minority groups acted to redefine “insider” and “outsider” status, or to alter their working environment by moving away from unhealthy or toxic environments or leaving the workplace altogether.

The way they were towards, eh, a, a Black, um, guy that was the admin. And he actually left a couple of weeks before I left ‘cause he was the one who mentioned it first. But I didn’t say it like that. He was like, do you notice they’re, like, racist on this ward? I don’t want to work here. I’m going to be… I’m going to a different ward in a couple of weeks.

*Asian, non-migrant, student nurse/midwife*

As a newly qualified staff when I go to my, whatever job I’m going to go for, if I don’t like the culture there, I’ll just move on and move on and move on and move on until I get a, find a team and culture where I do feel as I fit in.

*Asian, non-migrant, student nurse/midwife*

The way you pronounce things will be different from all people, but there are some among them, no matter how you say it, they wouldn’t understand you […] And the way they will behave or squeeze their face, you know it will have an impact on you, it will be down on you […] And in most cases when you come um, across people from the minority, actually, you enjoy more because they all understand you.

*Black, migrant, qualified nurse/midwife*

Reflecting examples of ethnic hierarchies and inter-ethnic group tensions, this segregation process also occurred between different racial and ethnic groups.

When I’m searching, I would like to go to a place that I find only White people, European, that’s fine. European, that’s fine. Usually, it’s African Black or Caribbean. And even if you search on YouTube and you see, like, women harassing Muslim women or like, most, 90 percent they are Black women.

*Other ethnic group, migrant, qualified nurse/midwife*

In contrast, segregation may reflect a preference for working in an environment with other minority groups, experiencing it as more supportive than those dominated by a White majority group.

Now in my place I haven’t experienced it [discrimination] per se, in my place, yeah because um, we are in team of the majority are from the minority group. So, even our manager is also from the minority group. And the other members, and the cooperation out there, and then how they support each other. Yeah. You wouldn’t experience it per se or do it, but let me put it this way, we are all the same. A few are um White, about two or three. The rest are from a minority. And the manager is also from the minority and welcomes everybody. We tell jokes and we talk to each other as if we are all equals.

*Black, migrant, senior qualified nurse*

Black African nurses especially in in-patient settings. I don’t find this is the case in community settings. I find it’s the only case in in um in-patient settings. In the community it’s very different it’s very diverse in terms of age, in terms of race and ethnicity. Um but in-patient settings […] because we, it’s a 24-hour care, so you pass on the responsibility to the next team, and I think you have that security as a nurse - you feel like the job is safer? So when you leave your shift, you’ve done your handover to the next team, who are your friends or people that you’ve worked with for years and years and years. They will always have your back.

*Asian, non-migrant, student nurse/midwife*

White participants often reported feeling excluded from these “cliques,” reflecting discomfort or fragility associated with the unfamiliar experience of exclusion as a minority.

I feel like, umm, the African nurses were kind of, were friends and they’d a-, also all been there a very long time, like they were all kind of friends in like a kind of clique and they like, I don’t know, they knew how to talk to each other, which sometimes to me felt like they were arguing but they weren’t they were just talking about something and then they’d laugh about it afterwards.

*White British, non-migrant, qualified nurse/midwife*

Potentially in opposition to the double jeopardy faced by migrant racial and ethnic minority groups, using shared (foreign) language could be a particularly powerful tool to invert predominant insider and outsider statuses and to subvert prevailing inequities in social support.

Like in the in the nursing office, sometimes they speak their own language, they - they just have this culture, I can’t explain it- they just have this culture about them? And. It just feels really uncomfortable and it feels like, you wouldn’t be supported, and I feel that’s one of the reasons why the nurses and the HCAs have been there for decades.

*Asian, non-migrant, student nurse/midwife*

#### Challenging racism

The features of workplace context described above also inhibited staff members capacity to challenge discrimination, bullying or harassment in turn serving to help maintain the status quo. Respondents described difficulties in challenging or reporting adverse experiences due to concerns about impact on career progression, fears of upsetting team dynamics and “rocking the boat,” and of being labelled a “snitch” or “troublemaker.” Such fears were reported by staff across racial and ethnic groups though for minoritised (particularly Black) staff, these concerns were weighted more heavily as they were compounded by the effects of prejudice and discrimination.

We know that particularly BME staff in particular won’t speak up. They are very reticent about speaking up about things that happened to them which is a concern[....] because people feared the consequences for themselves, they fear being called a snitch.

*Black, migrant, senior qualified nurse/midwife*

One of the midwives I was speaking to, she’s African, Black African, and she said to me that… I said to her, like, you need to make a complaint because there’s a couple of things that, that were being said about her at work. About her being difficult and stuff. And she goes, “oh, yeah, oh, yeah, and then I escalate it and you think that that, that stigma’s going to be taken away from me or get worse?”

*White British, non-migrant, qualified nurse/midwife*

## Discussion

Within racially and ethnically diverse healthcare settings, we qualitatively identified how the racialised organisational culture was shaped by and perpetuated micro-level inter-staff experiences of discrimination, bullying and harassment. Despite high diversity, inherently racialised hierarchical inequities reflected and influenced lack of inclusion. Bullying, prejudice, discrimination and harassment were used to exploit and maintain the racialised hierarchy, and were exacerbated in pressurised environments. Moreover, race, ethnicity, migration status, language and religion were among salient intersecting markers of similarity/dissimilarity. These processes stifled inclusion and often employed bullying or microaggressions. For racial and ethnic minority staff, ingroup maintenance could also be a way of coping with workplace bullying, discrimination and harassment, though the workplace context served to limit staff capacity to challenge and report unfair treatment.

### Organisational culture, high diversity and low inclusion

Participant accounts portrayed a highly racially and ethnically diverse but often un-inclusive workplace, and evidence suggests that where inclusivity is low, high levels of workforce diversity have been associated with racial tensions, conflict, as well as reduced group identity and employee cohesion, performance and satisfaction (Jackson & Joshi, 2011). Mannion and Davies (2018) described three layers of organisational culture inhibiting inclusion applicable to our findings, which helps illuminate why initiatives aiming for “culture change” are harder to implement than procedural changes (Scott et al., 2003). “Visible manifestations” of culture are artefacts reflecting “the way things are done here” (e.g. unequal distribution of labour, reward and progression opportunities). “Shared ways of thinking” are the values, beliefs and arguments used to justify and maintain these visible manifestations (e.g. unspoken assumptions about relative power status and value); and “deeper shared assumptions” are unconscious and unspoken expectations and assumptions underlying clinical practice and staff interactions (e.g. accepted behavioural norms around work/communication styles, unchallenged stereotyping). In support of Critical Race Theorist Ray’s (2019) “Theory of Racialised Organisations,” our findings suggest that these layers of organisational culture are inherently racialised, serve to limit the agency of racial minority groups (e.g. in pursuing promotion and challenging racism), legitimise the unequal distribution of social and material resources and promotes Whiteness as a credential. Our findings also indicate that these processes influence the maintenance of racialised hierarchies operating both vertically and horizontally, including between racial and ethnic minority staff groups. To complement our findings, future studies should explore the experiences of workplace pressures across racial and ethnic groups, and how this impacts racialised hierarchies.

### Coping with and challenging discrimination

Generally protective for mental and physical health, coping refers to the behavioural and cognitive processes used to manage demands appraised to exceed an individual’s resources (Lazarus & Folkman, 1984). Coping with racism has been proposed to require specific skills beyond those generally identified for stress and stigma including protecting the self (physically and psychologically), engaging in self-control and confronting racism (Mellor, 2004). Coping with racism has also been linked to strong racial identity, eliciting social support, and anger suppression and expression, with each associated with both positive and deleterious effects for individuals’ health and mental health (Brondolo et al., 2009). Our findings indicate that staff tended to cope individually and collectively by acting to protect themselves psychologically by engaging in ingroup behaviours, leaving for more supportive environments or leaving the health service, while opportunities to confront racism and to promote positive racial identity were inhibited by prevailing organisational culture as reflected in our findings about the barriers to reporting discrimination.

### The role of Whiteness

Ray (2019)’s Theory of Racialised Organisations and Moore (2008)’s Theory of “White Institutional Space” posit that Whiteness shapes institutional and organisational processes and thus Whiteness is seen as normative and neutral. White staff not only reinforce but also benefit the most from the unmarked Whiteness of organisations and racialised hierarchies (Moore, 2008; Ray, 2019). In our study, when asked about experiences or witnessing of discrimination, White British staff more commonly spoke about experiences of discrimination, bullying or harassment at work in relation to age, parenthood, seniority level or sexual orientation and gender identity. As demonstrated by some of the indicative quotes cited here, when White staff did comment on race and racism, they often either spoke in ways that minimised or denied experiences of racialised bullying on racially minoritised staff, attributed negative experiences to personality rather than race, expressed discomfort when they were the minority in a particular context or acknowledged discrimination but did not discuss action to challenge this. This may reflect the privilege they hold existing in a White organisation and further research is essential to highlight the role that majority White staff (and White institutional norms) have in maintaining racialised inequities (Link & Garcia, 2021).

### Tackling racial and ethnic workplace and health inequalities

Our findings strongly suggest that tackling bullying, discrimination and harassment and redressing occupational, and subsequent health inequities, necessitates disrupting accepted norms of organisational culture shaping staff interactions. This is challenging because of the complexity of the health service and the diversity of its workforce, intensity of staff-to-staff interactions and task interdependence, unequal distribution of power and disproportionate representation of racially minoritised staff at lower-banded levels. Further, this is set in the context of pervasive prejudice and stereotyping, and the inhibiting effects of anticipated discrimination and “racial battle fatigue” (Smith et al., 2007). This is compounded by lifetime experiences of discrimination both in- and outside work which undoubtedly has impacted the health and wellbeing of healthcare staff (Rhead et al., 2021; Todorova et al., 2010). For example, our term “insidious dismissal” describes the processes that drive some to move to other teams for more support and safety, often where there is greater ethnic minority representation and leadership. This subtle and often racialised process is distinct from “constructive unfair dismissal,” where an employer commits a serious breach of contract, entitling employees to resign (ACAS, 2021).

Interventions tackling discrimination among healthcare staff have been reviewed (Hatch et al., 2021; West et al., 2015), finding little evidence that initiatives such as cultural competency and unconscious bias training are effective in reducing discrimination and lack of research assessing their impact on staff health and wellbeing. These may be enhanced by involving racial and ethnic minority staff in their development (Hatch et al., 2021), incorporating experiential, perspective-taking and reflective training, and promoting anti-racism (Came & Griffith, 2018), including promoting allyship, bystander intervention and positive action. Additionally, our findings suggest that attention be paid to intersectional experiences, and to when in- and outgroup processes may have protection functions for minoritised staff groups.

Our study supports existing strategic-level recommendations to promote systems-change, including sustained strategies transparently linked to senior-level accountability targeting organisational, workplace inter- and intra-personal levels (Naqvi et al., 2019; West et al., 2015). This necessitates buy-in from all leadership levels, openly prioritising workforce diversity, and resourcing specific initiatives to increase inclusion (Priest et al., 2015; West et al., 2015). This may require sanctions by governing and regulatory bodies (the Care Quality Commission in the UK) for failures to comply and progress.

### Strengths and limitations

Data were collected just prior to the COVID-19 pandemic in the UK, the killing of George Floyd and subsequent Black Lives Matter activity. Our findings therefore exemplify the marginalisation, victimisation and racialised inequities prevailing prior to these events (King et al., 2021; Ubele Initiative, 2020). As with other qualitative studies, we cannot claim generalisability. However, situating the study in London offers key insights into the research questions since it has the most racially and ethnically diverse healthcare workforce with the greatest proportion of migrants in the UK. Further, framing our findings within a critical race perspective, supports and refines existing workplace diversity and inclusion frameworks. Importantly, our study was developed with healthcare staff and interpretations have been refined with a racially and ethnically inclusive team of researchers and healthcare practitioners.

## Conclusions

Stereotyped and prejudicial views of racial and ethnic minority and migrant staff inhibit inclusion and underpin intergroup biases which limit workgroup performance, staff experience and ultimately staff health. Efforts to promote work/life balance, proactively foster inclusion by equitably valuing and esteeming difference in roles, attributes, knowledge and perspectives, and proactively levelling-up opportunities for career progression are structurally key for staff retention and improved health outcomes. To reduce disparities, interventions require systemic shifts to organisational culture through directly addressing overt prejudice, stereotypes and microaggressions and their impact on health.

## Figures and Tables

**Table 1:** Sample characteristics

	*n*	%
Gender		
Female	41	85
Male	7	15
Race/ethnicity		
Asian	5	10
Black (African/Caribbean/other Black)	13	27
White British	19	40
White Other	7	15
Any other group	4	8
Migration status		
Migrant	17	35
Non-migrant	31	65
Seniority		
Student nurse/midwife	21	44
HCA or nursing associate	6	13
Entry-level nurse/midwife (Band 5)	10	21
Mid or senior-level nurse/midwife (Bands 6–8)	11	23

*Note:* Characteristics have been grouped to preserve anonymity.
